# Temporal self-regulation theory: a neurobiologically informed model for physical activity behavior

**DOI:** 10.3389/fnhum.2015.00117

**Published:** 2015-03-25

**Authors:** Peter A. Hall, Geoffrey T. Fong

**Affiliations:** ^1^Faculty of Applied Health Sciences, University of WaterlooWaterloo, ON, Canada; ^2^Department of Psychology, University of WaterlooWaterloo, ON, Canada

**Keywords:** executive function, exercise, theoretical neuroscience, brain, behavior

## Abstract

Dominant explanatory models for physical activity behavior are limited by the exclusion of several important components, including temporal dynamics, ecological forces, and neurobiological factors. The latter may be a critical omission, given the relevance of several aspects of cognitive function for the self-regulatory processes that are likely required for consistent implementation of physical activity behavior in everyday life. This narrative review introduces temporal self-regulation theory (TST; Hall and Fong, 2007, 2013) as a new explanatory model for physical activity behavior. Important features of the model include consideration of the default status of the physical activity behavior, as well as the disproportionate influence of temporally proximal behavioral contingencies. Most importantly, the TST model proposes positive feedback loops linking executive function (EF) and the performance of physical activity behavior. Specifically, those with relatively stronger executive control (and optimized brain structures supporting it, such as the dorsolateral prefrontal cortex (PFC)) are able to implement physical activity with more consistency than others, which in turn serves to strengthen the executive control network itself. The TST model has the potential to explain everyday variants of incidental physical activity, sport-related excellence via capacity for deliberate practice, and variability in the propensity to schedule and implement exercise routines.

## Temporal Self-Regulation Theory: A Neurobiologically Informed Model for Physical Activity Behavior

Physical activity promotion is of central interest to the fields of medicine and public health, as the prevention of chronic disease occurrence (primary prevention) and management of early onset (secondary prevention) both rely on it. Yet in order to increase physical activity behavior in the interests of disease prevention, it is necessary to have a firm understanding of the causal determinants of physical activity behavior itself. Such understanding in turn relies on iterative cycles of theory building, empirical testing and continued theory refinement. Our current theoretical models of physical activity behaviors largely mirror those of other health related behaviors, and have been drawn from applied social psychology, and to a lesser extent, clinical psychology and public health. Among the most well-known theories of physical activity behavior are the Transtheoretical Model (TTM; Prochaska et al., 1992) and the Theory of Reasoned Action/Planned Behavior (TPB; Ajzen and Madden, 1986).

Despite their initial popularity and apparent pragmatic appeal, these models have been criticized on a number of fronts, including their conceptual and epistemic foundations, empirical evidence base and ultimate testability (see Sutton, 1998; Ogden, 2003; West, 2005). Beyond these well-articulated criticisms, there is reason to believe that all of these models are also incomplete due to their non-inclusion of several categories of influence on difficult-to-implement behaviors such as physical activity. For instance, a substantial body of findings has emerged over the past decade or two regarding ecological influences on physical activity behavior (Sallis et al., 2006), as well as behavioral economic literatures highlighting the importance of temporal dynamics in decision making about behaviors that involve endurance of inconvenience or other costs, despite long-term cumulative benefits (Ainslie, 2013). Relatedly—and of most central consideration here—neurobiological aspects of self-regulatory processes are omitted entirely from both historic and contemporary models of physical activity behavior that have dominated the theoretical landscape in recent history. A growing body of literature, for example, identifies executive function (EF) as an important determinant of behaviors that require effort, consistency, and suspension of competing default behaviors (Hinkin et al., 2002, 2004; Hall et al., 2006; Insel et al., 2006; Hofmann et al., 2009, 2012; Allan et al., 2010; Nederkoorn et al., 2010; Stilley et al., 2010; Hall, 2012; Lowe et al., 2014). Effort, consistency and suspension of default preferences also seems characteristic of habitually implemented physical activity behavior, yet none of the current models include neurobiological factors that would theoretically facilitate the above.

## Executive Function in Health Behavior Theory

EF can be defined as a set of cognitive processes that serve to enable reflective, “top-down” (as contrasted with reflexive, “bottom-up”) control over behavior, thought and emotion, arising from the operation of distributed brain networks, with important nodes in the prefrontal cortex (PFC; Shallice and Burgess, 1993; Baddeley, 1996; Miller, 2000; Miyake et al., 2000; Miller and Cohen, 2001; Hofmann et al., 2012; Miyake and Friedman, 2012). The PFC is implicated in each of the three major subcomponents of EF, including working memory (i.e., the ability to work with finite information in an online state), inhibition (i.e., the ability to suspend prepotent responses), and mental flexibility (i.e., the ability to efficiently adapt to changing performance rules). Perhaps even more important than its status as a central (though non-exclusive; Van der Werf et al., 2003; Bellebaum and Daum, 2007) node in the executive control network is the PFC’s extensive connections with other centers that drive more reflexive processes, thus enabling effective modulation of such centers more directly than would otherwise be possible (Miller and Cohen, 2001; Tekin and Cummings, 2002).

**Figure 1:**
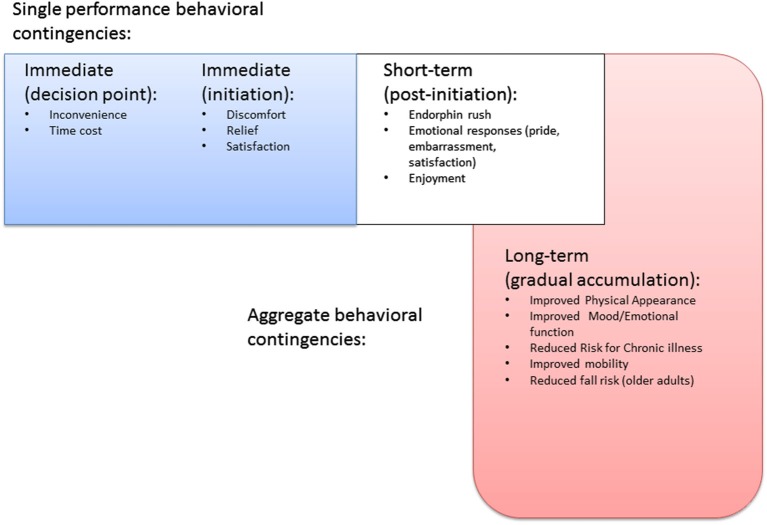
**Timeline of behavioral contingencies for physical activity behavior**.

For example, the PFC is extensively interconnected with reward centers of the brain, which are evolutionally much older and more primitive than the neocortex (Groenewegen et al., 1997; Tekin and Cummings, 2002). This interconnectedness is especially important when explaining the potential for humans to forgo momentary pleasure, or reflexive reactions to the immediate environment, in the interests of other benefits that might be more long term in nature (Miller and Cohen, 2001; Hare et al., 2009; Figner et al., 2010; Luo et al., 2012). The ability to participate in physical activity behavior repeatedly for the sake of non-immediate benefits (i.e., disease/disability prevention, appearance or emotional benefits not manifest at the time of performance) is arguably one of the more important aspects of physical activity execution. While the experience of engaging in physical activity may be pleasurable, the lead up to initiation of each bout often involves forgoing more hedonically pleasurable activities (see Figure 1), and for new initiators, even the exercise experience itself may not be especially pleasurable until sufficient aerobic/muscular fitness has been built up, and until a certain level of confidence in one’s abilities has been reached (Hall and Fong, 2007). Even activity in the form of sport participation that is experienced as “fun” by participants requires endurance of significant exertion, time cost, and even outright discomfort. Competitive sport, arguably even more so, requires commitment to endurance of long hours of practice not experienced as pleasurable by any except the most masochistic. Indeed, some argue that it is the capacity for such practice that may explain success in competitive sport more so than innate ability (Ericsson et al., 1993; Ericsson, 2014).

Beyond forgoing immediate pleasures, there are some specific benefits enabled by each of the facets of EF that are relevant to maintaining patterns of difficult-to-implement behaviors such as physical activity. First, the ability to keep activity goals in mind during decision making, or while engaged in other tasks, is enabled by working memory. Second, those with stronger inhibitory capacities may be better able to remember and implement physical activity plans in place of more compelling or immediately rewarding activities. And finally, mental flexibility may facilitate flexible adaptation of such plans to changing circumstances throughout the day, week or month, thereby enabling more consistent physical activity participation in the long run. Of the three facets of EF, arguably inhibition is the most central (Miyake and Friedman, 2012). Behavioral inhibition can be defined as the capacity to suspend prepotent responses to stimuli, and enables the possibility of behaving in ways that are counter to habit, visceral impulse, or social norms. For sedentary individuals, having relatively strong EF may be required to behave in active ways despite habitual non-activity, despite the appeal of maintaining inactivity, and contrary to peer and family norms which may reinforce inactivity directly or indirectly.

More importantly, there is potential for EF and physical activity to be functionally connected through positive and negative feedback loops over time. There is now a substantial literature of experimental studies demonstrating that participation in physical exercise improves EF (Kramer et al., 1999; Colcombe and Kramer, 2003; Colcombe et al., 2004; Liu-Ambrose et al., 2010; Smith et al., 2010; Davis et al., 2011). Likewise, there is now literature supporting the potential for EF to reinforce physical tactivity participation; for example, a recent study by Best et al. from this section found that participants in structured exercise programming were more likely to adhere to physical activity following conclusion of the structured programming if they experienced EF gains during the intervention period (Best et al., 2014). Likewise, Daly et al. showed time lagged associations between EF and physical activity participation that support the contention that they are mutually reinforcing over time (Daly et al., 2015). Finally, McAuley et al. (2011) demonstrated that EF predicted exercise class attendance via self-efficacy beliefs in the context of an exercise trial over the course of a 1-year interval among older adults. Together these studies provide preliminary evidence supporting the existence of positive and negative feedback loops involving physical activity and EF strength.

Given the conceptual role that EF plays in self-regulatory processes involved in repeated participation in physical activity over time, it seems sensible that it should have a role that is fairly central in theoretical models for physical activity behavior. The inclusion of EF in turn has the benefit of linking physical activity promotion—and the broader enterprise of health behavior change—with the larger human neuroscience literature on self-regulatory processes (Hofmann et al., 2012; Ochsner et al., 2012).

## Temporal Self-Regulation Theory

**Figure 2:**
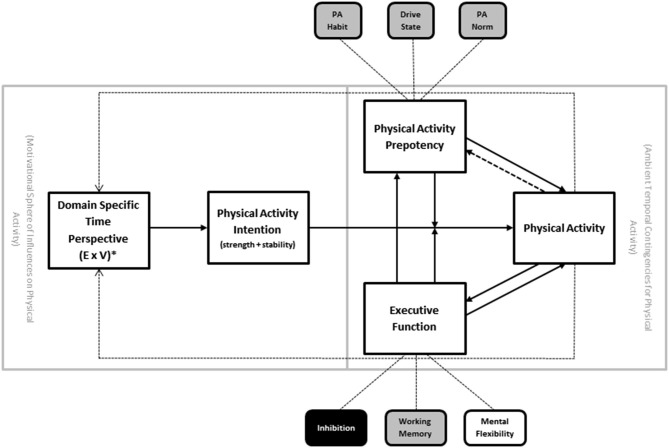
**Temporal self-regulation theory for physical activity (TST-PA)**. Note: PA = physical activity. Solid arrows indicate major causal pathways; dotted lines represent secondary causal pathways representing cumulative effects over time. * = temporally weighted.

Temporal self-regulation theory (TST; Figure 2) was introduced to account for the limitations of some of the existing social cognitive models of health behavior, including the lack of neurobiological factors such as EF (Hall and Fong, 2007, 2013). Briefly, the model posits that there are three proximal determinants of behavior: intention, prepotency and EF. The proximal model is in turn modulated by the ecological context, such that EF and prepotency become increasingly important causal links under conditions where the behavior is performed in an unsupportive environment (i.e., where there is large disjunction between when the costs and benefits for the behavior are incurred). In the absence of such temporal disjunction—a purely theoretical state of affairs that rarely exists for health related behaviors—the intention-behavior link would be assumed to be uniform and not moderated by prepotency or executive control (i.e., intentions are perfectly translatable into behavior). Intention in turn is determined by beliefs and values attached to the behavior based on many sources, including personal history with the behavior, but also other exogenous forces.

## Proximal Model

One of the central facets of the TST model that it retains from earlier social cognitive models is the presence of intention as determinant of physical activity behavior. However, it is thought that the intention behavior link is subject to modification by both EF and behavioral prepotency (see above). Each of these has substantial empirical precedent from the experimental research literature (Webb and Sheeran, 2006; Jansen et al., 2013). Prepotency of physical activity behavior could be influenced by the extent to which physical activity is considered to be normative in the social environment (“norm”), strength of established habit to be either active or inactive (“habit”), or drive states that either facilitate or interfere with physical activity (“drive”). In the case of physical activity (as opposed to other behaviors) it is assumed that norm and habit are stronger determinants of prepotency than drive states (whereas drive state may indeed be more influential for other behaviors that are more viscerally determined, such as substance use and dietary behavior).

## Feedback Loops

Other facets of the proximal portion of the model are the addition of recursive feedback loops between physical activity behavior and both prepotency and EF. The recursive links back to prepotency are thought to be driven by the gradual cementing of physical activity habit strength and norm enhancement that would occur with repeated physical activity performance over time. The recursive link back to EF is based on the now extensive literature linking exercise training to enhancement of brain regions that support EF (Colcombe and Kramer, 2003; Smith et al., 2010), and more recent studies document the relationship between EF and physical activity implementation (McAuley et al., 2011; Best et al., 2014; Daly et al., 2015). Of additional interest is the link between EF and prepotency, reflecting the potential of strong EF to derail norm and habit driven inactivity in the interests of introducing physical activity behavior despite low behavioral precedent.

## Contextual Modeling

Ambient temporal contingencies are the final component of the proximal model, and these reflect the degree of temporal disjunction between costs and benefits of a behavior imposed by the ecological context in which the behavior occurs. The higher the temporal dispersion of costs vs. benefits, the greater the requirement for self-regulation. For instance, examples of physical activity that include many immediate costs (e.g., inconvenience, time costs, monetary costs) but very gradual or delayed benefits (e.g., improved appearance, acceptance from peers, improved mobility), would tend to rely on self-regulatory processes involving EF, intention, and prepotency. Those instances of activity that have costs and benefits that fall around the same point in time might be more pure manifestations of simple decision making rather than self-regulation *per se* (e.g., the decision to join a game of basketball while already on the court with no competing time requirements). Because of the nature of physical activity and the range of ecological environments in the modern world, it is assumed that on balance there is a temporal dispersion of costs and benefits for activity, such that costs are relatively immediate and benefits are delayed or accumulate gradually after repeated performance. Different ecological parameters can amplify or reduce this temporal dispersion, by removing barriers or increasing ease of access to physical activity performance opportunities, for example.

**Figure 3:**
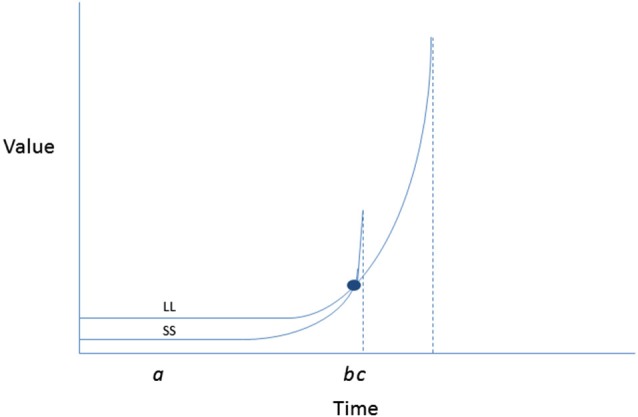
**Inverted hyperbolic discount curves illustrating a preference reversal involving forgoing larger later (health benefits, improved appearance) for smaller sooner (convenience, indulgence) contingencies when choosing between exercise and a competing enjoyable activity, such as video game playing**. The y axis depicts subjective value of a reward associated with each of the activities at each time point. The *x* axis depicts the relative immediacy of receipt of each reward. At point a when both rewards are removed in time, the LL rewards of physical activity are preferred (i.e., have higher subjective value) than the SS rewards of computer game playing. However, with passage of time as SS becomes more immediately available, a spike in value is experienced. At b an indifference point is reached at wherein the value of SS (video game playing) and LL (exercising) are equivalent; at point c, with video game playing immediately available, its subjective value surges past that of exercise (despite having been initially less preferred). Adapted from Ainslie (2013).

Further back in the model, TST proposes determinants of intention itself, which are thought to be reducible to temporally anchored perceptions of expectancy and value. The latter parameter is assumed to be hyperbolic in relation to time such that outcomes that are temporally nearer are of more value, with immediacy associated with a very sharp spike in value (Figure 3, adapted from Ainslie, 2013). This hyperbolic shape to the value curve explains the tendency for preference reversals to occur (i.e., the tendency to make resolutions to be active on New Year’s Eve, but then when facing the time to attend the first fitness class, preferring a competing sedentary activity instead).

## Comparing and Contrasting TST with Existing Models

There are a few features that TST shares with existing models. First, there is a role for intention as a determinant of behavior. This is one feature that TST shares in common with TPB, as well as—to some extent—Social Cognitive Theory (Bandura, 1986), which also posits motivation as an important determinant of behavior. Additionally, like TPB, there is an upstream role for beliefs about possible outcomes of behavior. Finally, like TPB, there is a control related variable, though it is not perceived control *per se*, but rather, neurobiologically-based control resources that emanate from the operation of the PFC and its interconnectivity with more reflexive brain centers (the striatum and the limbic system).

Points of departure from existing models include the explicit use of temporal proximity as a determinant of the potency of both behavioral outcomes that are anticipated cognitively, as well as the relative influence (in a strict behaviorist sense) of contingencies encountered in the ecological context in which physical activity is performed. Such temporal contingencies (imagined and real) are central to the TST model vis-à-vis setting the stage for self-regulatory processes that take over where decision making leaves off. It is further assumed by the TST that the shape of the value curve for behavioral outcomes (real and imagined) is hyperbolic, as per Ainslie (2013). This is a well documented phenomenon, which explains the tendency for individuals deciding between two options (a smaller sooner (SS) reward and a larger later reward (LL)), to revert from preference for the LL reward in favor of the SS reward when the latter becomes imminent.

The dynamics of these choices refer to the nature of the values attached to each alternative. Essentially the preference reversal potentiates behavior that is counter to initially formed intention as the availability of the tempting alternative becomes more imminently available. In terms of physical activity, there are certainly many competing alternative behaviors, only one of which is video game playing. This however, is a highly prototypic example, given that these two behaviors do seem to compete especially in younger adults and children (Barnett et al., 2010).

Importantly, the preference reversal described above does not necessitate a behavioral reversal, but merely makes it more likely, given the partial dependence of behavior on value/preference. Active self-regulation counter to currently experienced value change amounts to active self-regulation, and within the TST model, can be withstood with enough behavioral precedent, and with enough executive control. That is, both prepotency of prior exercise experience and well developed EF can maintain the stability of intention to exercise in the presence of the immediately available, and more tempting alternative behavior. Indeed studies have shown that choosing the LL reward over the SS reward is associated with greater activation in the prefrontal areas of the cortex that support EF (Luo et al., 2012).

## Applying TST to Model Physical Activity Promotion

**Figure 4 F4:**
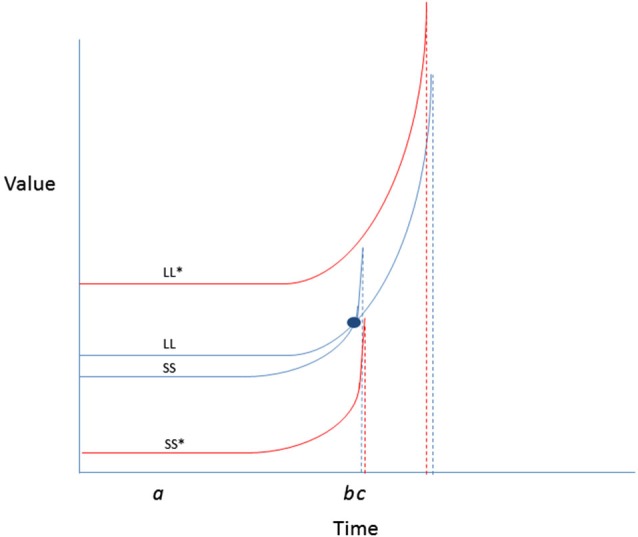
**Two possibilities for behavior change are presented: enhancing LL value or reducing SS value change.** Enhancing the values attached to physical activity outcomes (by increasing salience, or making them more vivid) could raise the value curve (LL*) such that it no longer crosses the SS curve. Likewise, reducing the value of video games (by making them more difficult to access, or less normative) until the window of availability passes, could reduce temptation. Simultaneous enhancement of LL and reduction of SS could be the most efficient means for reducing likelihood of preference reversal in favor of a competing sedentary behavior.

There are several maxims that can be derived from TST when applied to physical activity promotion, some of which align with current best practices, and others that are more novel[Fig F4]:

1. *Improve temporal balancing of costs and benefits associated with exercise*. Generally, physical activity performance brings immediately felt costs (time, convenience), and gradual accumulation of benefits (appearance, health). For some, the experience of performance is also pleasurable, once initiated. Amplifying these pleasurable aspects of experience, and making them salient at choice time may be helpful. Likewise, some categories of immediate cost can be mitigated; for instance, removing or reducing the monetary cost associated with activity (for instance by subsidizing facility memberships) may assist inactive people in need to become more active, and be guided by non-immediate benefits. Improving accessibility of venues and equipment necessary to exercise may also provide benefit in this respect.
2. *Optimize executive function*. Removing the influence of agents that reduce EF, such as sleep deprivation, alcohol and stress may serve to optimize the cognitive control mechanisms that are essential for implementing physical activity plans that have already been formed. Once activity begins in earnest, there could be a feed forward benefit of physical activity on EF that might render subsequent activity implementation more consistent, and less draining of personal resources.
3. *Induce intention strength and stability* by emphasizing, and rendering salient at crucial choice points, the expected cumulative benefits of physical activity. Often these are known intellectually in a remote sense, but are crowded out by other thoughts at times when decisions about activity are being made.
4. *Amplify valuations* and expectancies for exercise, and mitigate costs. Although the hyperbolic discount curves that characterize outcomes as a function of immediacy may suggest that rewarding sedentary activities may be more valued, there are some possible ways of improving the expectancy-value landscape for energy burning forms of physical activity. Some of the theoretical possibilities are represented in Figure 4. As depicted in the example, encouraging greater spread in value curves separating exercise from a competing alternative sedentary behavior could reduce the likelihood of preference reversals in favor of the latter.
5. Encouragement of physical activity requires *multi-level action*, ranging from policy and built environment to individual behavior change in order to facilitate implementation, in much the same way we need to exploit these same routes for other behavioral imperatives for disease prevention (Marteau et al., 2012; Marteau and Hall, 2013).

## Summary

TST conceptualizes physical activity behavior as being centrally influenced by intention strength/stability, behavioral prepotency and EF. The latter is particularly central, and yet it has not previously been incorporated into any formal model for physical activity behavior. The TST model hypothesizes mutual reinforcement of EF and physical activity over time, a self-regulatory loop supported by several empirical studies in this section. Research supporting both the implementation-facilitating effects of EF on other difficult-to-implement health behaviors, and the effects of physical activity on brain systems that support EF, are both buttressed by findings from individual experimental studies and, in some cases, meta-analytic summaries of such work. Future research may inform the specificity of the TST model in relation to physical activity by identifying critical thresholds of EF and physical activity required to achieve self-perpetuation of the EF-activity cycle over time. Establishing such thresholds would constitute a key step in establishing the required prescriptive dose to enhance likelihood of long-term maintenance of physical activity behavior in the general population.

## Conflict of Interest Statement

The authors declare that the research was conducted in the absence of any commercial or financial relationships that could be construed as a potential conflict of interest.
